# Two genomic regions of a sodium azide induced rice mutant confer broad-spectrum and durable resistance to blast disease

**DOI:** 10.1186/s12284-021-00547-z

**Published:** 2022-01-10

**Authors:** Kuan-Lin Lo, Yi-Nian Chen, Min-Yu Chiang, Mei-Chun Chen, Jerome P. Panibe, Chung-Chun Chiu, Lu-Wei Liu, Liang-Jwu Chen, Chun-Wei Chen, Wen-Hsiung Li, Chang-Sheng Wang

**Affiliations:** 1 Department of Agronomy, National Chung Hsing University, Taichung, Taiwan; 2 Division of Plant Pathology, Taiwan Agriculture Research Institute, Taichung, Taiwan; 3 Institute of Molecular and Cellular Biology, National Tsing Hua University, Hsinchu, Taiwan; 4 Bioinformatics Program, Taiwan International Graduate Program, Institute of Information Science, Academia Sinica, Taipei, Taiwan; 5 Biodiversity Research Center, Academia Sinica, Taipei, 115 Taiwan; 6 Institute of Molecular Biology, National Chung Hsing University, Taichung, Taiwan; 7 Advanced Plant Biotechnology Center, National Chung Hsing University, Taichung, Taiwan; 8 Department of Ecology and Evolution, University of Chicago, Chicago, IL 60637 USA

**Keywords:** Rice blast, Sodium azide mutant, Broad-spectrum resistance, QTL-seq mapping

## Abstract

Rice blast, one of the most destructive epidemic diseases, annually causes severe losses in grain yield worldwide. To manage blast disease, breeding resistant varieties is considered a more economic and environment-friendly strategy than chemical control. For breeding new resistant varieties, natural germplasms with broad-spectrum resistance are valuable resistant donors, but the number is limited. Therefore, artificially induced mutants are an important resource for identifying new broad-spectrum resistant (*R*) genes/loci. To pursue this approach, we focused on a broad-spectrum blast resistant rice mutant line SA0169, which was previously selected from a sodium azide induced mutation pool of TNG67, an elite japonica variety. We found that SA0169 was completely resistant against the 187 recently collected blast isolates and displayed durable resistance for almost 20 years. Linkage mapping and QTL-seq analysis indicated that a 1.16-Mb region on chromosome 6 (*Pi169-6*(*t*)) and a 2.37-Mb region on chromosome 11 (*Pi169-11*(*t*)) conferred the blast resistance in SA0169. Sequence analysis and genomic editing study revealed 2 and 7 candidate *R* genes in *Pi169-6*(*t*) and *Pi169-11*(*t*), respectively. With the assistance of mapping results, six blast and bacterial blight double resistant lines, which carried *Pi169-6*(*t*) and/or *Pi169-11*(*t*), were established. The complementation of *Pi169-6*(*t*) and *Pi169-11*(*t*), like SA0169, showed complete resistance to all tested isolates, suggesting that the combined effects of these two genomic regions largely confer the broad-spectrum resistance of SA0169. The sodium azide induced mutant SA0169 showed broad-spectrum and durable blast resistance. The broad resistance spectrum of SA0169 is contributed by the combined effects of two *R* regions, *Pi169-6*(*t*) and *Pi169-11*(*t*). Our study increases the understanding of the genetic basis of the broad-spectrum blast resistance induced by sodium azide mutagenesis, and lays a foundation for breeding new rice varieties with durable resistance against the blast pathogen.

**Supplementary Information:**

The online version contains supplementary material available at 10.1186/s12284-021-00547-z.

## Background

Rice blast, caused by *Pyricularia oryzae* (syn. *Magnaporthe oryzae*), is one of the most destructive rice diseases and can completely ruin the yield of a rice field (Chen and Chen 2020; Wang et al. 2014). Fungicide application is a major strategy to control the blast disease, but utilization of fungicides is costly and produces pollution to the environment. To manage the blast disease, breeding resistant varieties is the most economic and sustainable strategy (Miah et al. 2013). However, since the blast pathogen genome evolves rapidly in the field, a resistant variety often loses its resistance within a few years (Huang et al. 2014). To prolong the durability of resistance, exotic germplasm, landraces, and wild relatives have been widely exploited to confer novel resistant (*R*) genes/alleles to local cultivars. However, introgression of an *R* gene/allele may undergo the problem of linkage drag, such that the undesirable traits cannot be removed even after generations of backcrossing (Ning et al. 2020; Singh et al. 2018).

Chemical/physical mutagenesis is another way to develop new *R* genes/alleles that do not exist in natural germplasm, and can generate desirable resistant mutations that are free from the association of undesirable genetic changes (Khan et al. 2009; Srivastava et al. 2017). Several mutagenic agents, including diethyl sulphate (DES), ethylmethanesulfonate (EMS), N-methyl-N-nitrosourea (MNU), sodium azide (SA) (chemical mutagens), fast neutron, and γ-ray (physical mutagens), have been utilized to generate novel mutants for improving rice blast resistance (Wang et al. 2019a). Some mutants even had achieved prominent success, providing significant economic contributions (Ahloowalia et al. 2004; Mohamad et al. 2006). For example, a γ-ray induced mutant R917 showed broad-spectrum resistance to the blast pathogen and have been used as a resistance donor in numerous breeding programs (Zhang et al. 2003). The other γ-ray induced blast resistant mutant Zhefu 802 was the most extensively planted variety during 1986–1994 in China, and its accumulative planted areas reached ~ 10.6 million ha (Ahloowalia et al. 2004; Shu et al. 1997).

A common strategy to develop durable blast resistant variety is to identify new *R* genes/alleles from either natural or mutation-derived germplasm, followed by the pyramiding of the *R* genes into a single variety through marker-assisted selection (MAS) (Joshi et al. 2019; Lei et al. 2013). To date, at least 118 *R* genes and about 350 QTLs for rice blast resistance have been mapped, and more than 30 *R* genes have been cloned (Ballini et al. 2008; Kalia and Rathour 2019; Mishra et al. 2021; Wang et al. 2017). Most of them were derived from rice cultivars and ~ 4% were derived from wild rice species (Ashkani et al. 2016). A number of blast resistant genes have already been identified from the induced blast resistant mutants, such as the *spl7* gene from a γ-ray induced mutant (Yamanouchi et al. 2002), the *LIL1* and *spl11* genes from EMS induced mutants (Zeng et al. 2004; Zhou et al. 2017), and the *blm* and *spl28* genes from MNU induced mutants (Jung et al. 2005, 2006; Qiao et al. 2010). Given that the genomes of numerous induced mutants are not well studied yet, many valuable *R* genes remain to be identified.

Sodium azide (SA) is a powerful mutagen and has been exploited to improve disease resistance in several plant species (Khan et al. 2009), including Panama disease tolerance in banana (*Musa* spp. AAA) (Bhagwat and Duncan 1998), mildew resistance in barley (*Hordeum vulgare*) (Molina-Cano et al. 2003), red rot resistance in sugarcane (*Saccharum officinarum*) (Ali et al. 2007), and bacterial blight and sheath blight resistance in rice (*Oryza sativa* L.) (Singh et al. 2020; Tseng et al. 2015). However, reported sodium azide induced rice blast resistant mutants are very limited, which include the Namil(SA)-bl5 mutant developed by National Institute of Crop Science (NICS) in Korea, and CRM 49 and CRM 51 mutants by ICAR-National Rice Research Institute in India (Cho et al. 2014; Singh and Singh 2018). Our previous study identified 50 sodium azide induced blast resistant/susceptible mutants from a mutation pool of a japonica variety TNG67 (Wang et al. 2019a). This study suggested that sodium azide is a potent mutagen to induce sufficient mutations throughout the genome that can efficiently generate diverse and broad-spectrum blast resistant mutants in rice (Wang et al. 2019a). To further identify valuable *R* genes in the sodium azide induced mutants for blast resistance improvement in breeding, one of the broad-spectrum blast resistant mutants, namely SA0169, was selected in this study. We focused on the resistance spectrum of SA0169 using the 187 blast isolates recently collected from 12 counties in Taiwan, and conducted the classical linkage mapping and QTL-seq analysis (Takagi et al. 2013) to determine the genomic regions associated with blast resistance. Moreover, candidate *R* genes in the mapped regions were identified. We also developed near isogenic lines (NILs) to validate the resistance spectrum of the mapped regions. In summary, this study successfully identified two complementary *R* regions in SA0169, revealing its durable and broad resistance spectrum, and also demonstrated the application potential of these two identified *R* regions for developing broad spectrum blast and bacterial blight double resistance lines.

## Results

### SA0169 exhibits broad-spectrum and durable resistance to blast

Our previous study showed that SA0169 conferred broad-spectrum resistance against all of the 89 Taiwanese blast isolates isolated during 1988–1991 (Wang et al. 2019a). SA0169 has been planted and showed resistance in our experimental field since ~ 20 years ago. To understand if SA0169 is resistant against blast isolates recently collected from fields, this mutant line was challenged with 187 newly collected blast isolates from 12 counties in Taiwan (2019). The results showed that SA0169 was resistant to all of the isolates tested (187/187, 100%; Additional file 1: Table S1), while other recently developed Taiwan varieties displayed a much lower resistance frequency (RF) (16.6% for Taikeng 14; 27.3% for Tainan 11; 34.2% for Tainung 71; 42.2% for Taikeng 9; Additional file 1: Table S1). Because of the rapid evolution of the blast fungal genome, a blast resistant population generally loses resistance within a few years. It is therefore remarkable that SA0169 showed resistance not only to the blast isolates collected in 1988–1991 but also to the isolates collected in 2019. Besides, SA0169 had kept blast resistance in the fields during the past ~ 20 years. These observations suggest that SA0169 contains valuable *R* genes. In order to utilize the blast resistance of SA0169 and to breed new disease resistant lines, we conducted (1) a genomic study of SA0169 and (2) disease resistance breeding, simultaneously. The mapped *R* regions by genomic study assisted the disease resistance breeding, while the various NILs (near isogenic lines) developed by the breeding program supported the genomic study of SA0169. The detailed results and the combinatory strategy are described below:

### Genomic study of SA0169

**Fig. 1:**
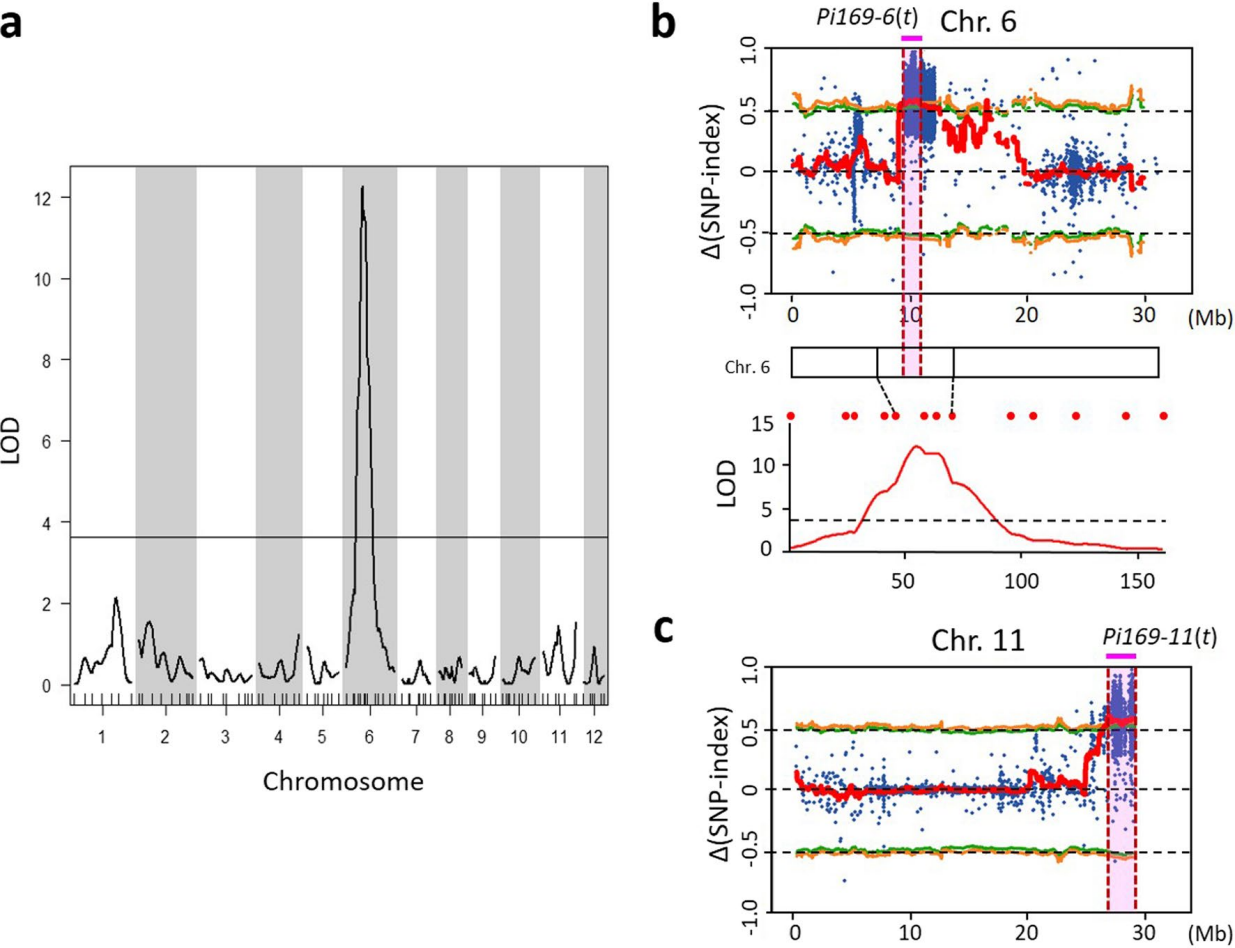
Mapping of the blast resistance genes in SA0169 using linkage analysis and QTL-seq. **a** Log of odds (LOD) plot by the Haley-Knott regression method. Solid line indicates the LOD threshold of 3.64. **b** Δ(SNP‐index) plot of chromosome 6 with statistical confidence intervals under the null hypothesis of no QTLs (green lines, *P* < 0.05; orange lines, *P* < 0.01). Blue dots, ∆ SNP‐index. Red lines, the average Δ(SNP‐index) in a 1 Mb region using a 10 kb sliding window. Pink shaded region indicates the candidate region of blast resistance using the QTL-seq strategy (*Pi169-6*(*t*)). Bottom, LOD plot of blast resistance by linkage mapping. **c** Δ(SNP‐index) plot of chromosome 11. Pink shaded region indicates the candidate region of blast resistance (*Pi169-11*(*t*))

To map the genomic regions associated with the blast resistance in SA0169, a highly blast susceptible line LTH (Lijiangxintuanheigu), which showed susceptible responses to 175 of the 187 isolates, was crossed with SA0169 to establish the F_2_ population for genetic analysis and mapping. A total of 122 F_2_ offspring of LTH×SA0169 were challenged with the blast isolate MS2a2-1209 and the segregation of F_2_ progenies fitted the 3:1 ratio of resistance to susceptibility, indicating that the blast resistance of SA0169 may be controlled by a single dominant locus (*χ*^2^ = 0.10, *P* = 0.75) (Wang et al. 2019a) (Additional file 2: Table S2). To map the *R* loci of SA0169, a total of 1,102 simple sequence repeats (SSR) markers were applied to genotype the two parental lines, LTH and SA0169. Most of the 1,102 markers tested showed no polymorphism; only 104 markers revealed sufficient reproducibility and stability for distinguishing between the genotypes of the two parents. A genetic map was constructed with these markers with 93 F_2_ individuals by the R/qtl software. These markers distributed throughout the 12 chromosomes with an average distance of 16.4 cM and the sum of the genetic distance of the 12 chromosomes is 1,504.6 cM. The results further showed that the confident region from 52 to 65 cM on the short arm of chromosome 6 with a LOD score of 12.3 at the peak and accounted for 45.5% of the phenotypic variation (Additional file 3: Table S3 and Fig. 1a, b). These results suggested that one major locus may largely account for the blast resistance of SA0169. In summary, both the genetic analysis and coarse mapping suggest that one locus on the short arm of chromosome 6 is largely responsible for the blast resistance in SA0169.

To verify the results of linkage mapping, we applied the QTL-seq method (Takagi et al. 2013). The blast susceptible parent TNG67 was crossed with the resistant mutant SA0169 and an F_2_ population was established. A total of 968 F_2_ offspring were inoculated with the blast isolate MS2a2-1209, and the segregation of blast response does not fit to 3:1, or 15:1, suggested that there may be more *R* genes, instead of one or two dominant genes controlling the blast resistance in SA0169 (Additional file 2: Table S2). To map the QTLs in SA0169, 18 F_2:3_ individuals from each F_2_ plant (a total of 155 F_2_ offspring) were inoculated with the blast isolate MS2a2-1209 and their blast responses were investigated. The 20 F_2_ plants that showed the highest proportion of resistant F_2:3_ progenies were selected as the R (resistant) group, while the 20 F_2_ plants that showed the highest proportion of susceptible F_2:3_ progenies were selected as the S (susceptible) group (Additional file 4: Table S4). The DNAs from these two groups were extracted and pooled to represent the ‘R’ and ‘S’ bulks, respectively. The two DNA bulks and their parents’ DNA were subjected to whole-genome resequencing. The Illumina high-throughput pair-end sequencing of the two DNA bulks generated average 166,553,526 high-quality sequence reads with an average of ~ 67.28-fold coverage depth and ~ 98.77% genome coverage (Additional file 5: Table S5). The comparative genome sequence data analysis between the parent TNG67 and the two bulks identified 42,422 high-quality nucleotide variants. For each variant, the SNP-index and Δ(SNP‐index) were calculated. The average Δ(SNP‐index) with 1 Mb window size and 10 kb increment were plotted across the genomic locations of the reference Nipponbare genome (Additional file 10: Fig. S1). We identified two genomic regions that exhibited high Δ(SNP‐index) values, which were correlated with the blast resistance. The first one is on the proximal region of the short arm of chr. 6 from 9.63 to 10.79 Mb (about 1.16 Mb long) with an average Δ(SNP‐index) = 0.56 (statistically significant under the null hypothesis: *P* < 0.01), which corresponded to the genomic region identified by linkage analysis (Fig. 1b). The other region is on the distal region of the long arm of chr. 11 from 26.65 to 29.02 Mb (about 2.37 Mb long) with an average Δ(SNP‐index) = 0.57 (*P* < 0.01) (Fig. 1c). In this paper, the first genomic region is designated as *Pi169-6*(*t*), and the second one as *Pi169-11*(*t*).

**Fig. 2:**
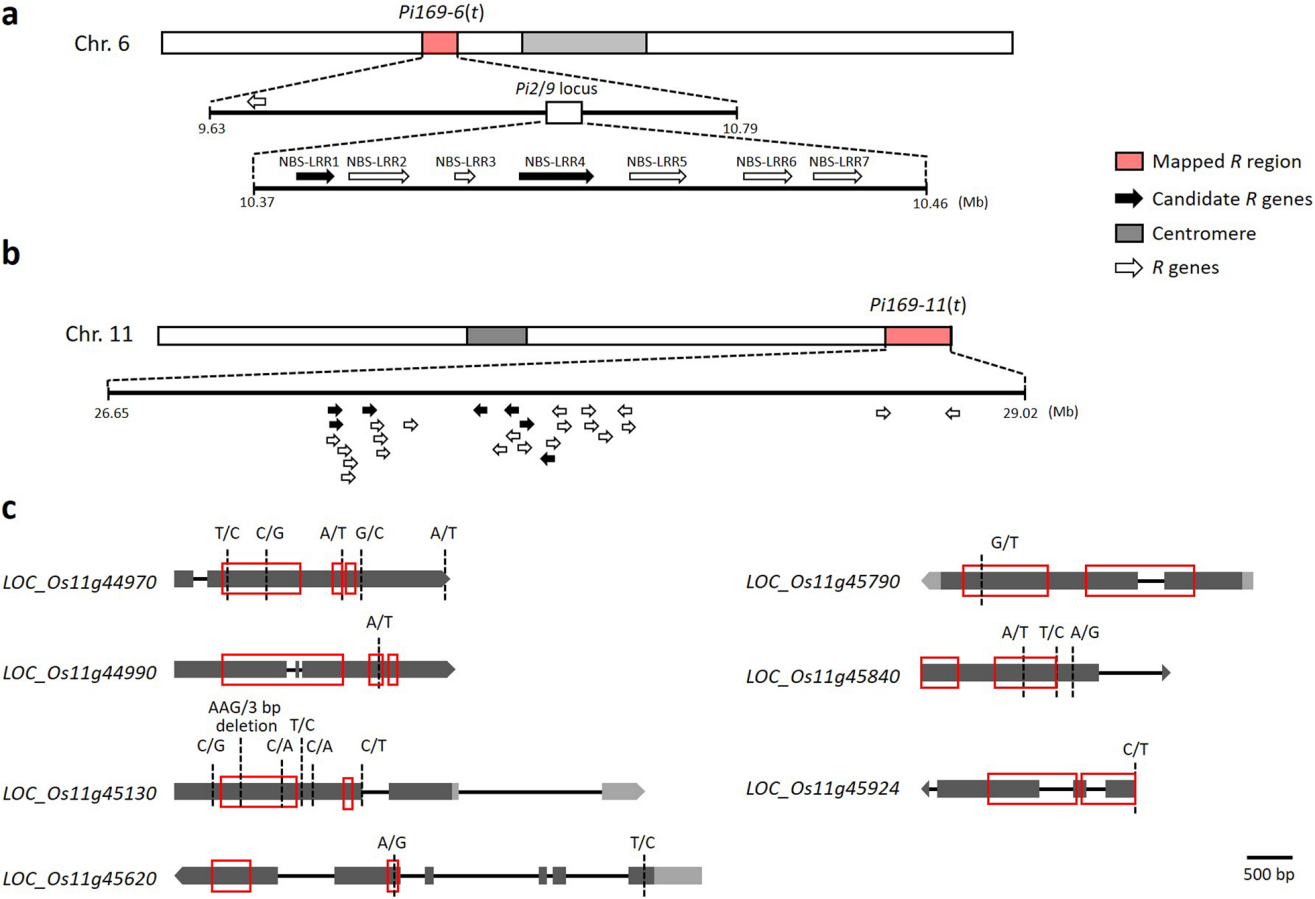
Identification of candidate *R* genes in *Pi169-6*(*t*) and *Pi169-11*(*t*) in SA0169. **a** The *R* candidates identified in *Pi169-6*(*t*) on chromosome 6. **b** The *R* candidates identified in *Pi169-11*(*t*) on chromosome 11. Pink shaded region: the candidate region associated with rice blast resistance. Due to space limitation, the size of *R* genes was not scaled. **c** The sequence analysis of 7 candidate *R* genes in *Pi169-11*(*t*). Red boxes, the predicted functional domains

To identify the candidate genes conferring blast resistance in SA0169, the annotated *R* genes within the confidence region *Pi169-6*(*t*) (9.63–10.79 Mb; 1.16 Mb) was surveyed. The *Pi169-6*(*t*) region includes 68 genes with 147 non-synonymous mutations. Of these 68 genes, six (NBS-LRR1, NBS-LRR2, NBS-LRR4, NBS-LRR5, NBS-LRR6 and NBS-LRR7; Fig. 2a) have been reported as *R* genes and all were in the *Pi2/9* locus (Zhu et al. 2012). Of the 6 genes, NBS-LRR5, NBS-LRR6 and NBS-LRR7, are partial (pseudo-), or non-functional genes; NBS-LRR1, NBS-LRR2 and NBS-LRR4 have a complete gene structure and so might be associated with the blast resistance in SA0169 (Luo et al. 2012; Zhu et al. 2012). Notably, NBS-LRR2 is also known as *Pi9*, which showed broad-spectrum resistance to blast pathogens in many countries and has been utilized in numerous breeding programs (Liu et al. 2002; Luo and Yin 2013; Ni et al. 2015; Qu et al. 2006; Wang et al. 2015). To further clarify the genomic structure, one *Pi9* specific primer pair was used to clone the *Pi9* gene in SA0169 (*Pi9-169*; GenBank accession number MZ327710) and its parent TNG67 (*Pi9-T67*; MZ327711). The cloned sequences confirm the SNP calling results: in TNG67 the *Pi9-T67* allele has a premature stop codon TGA at positions 298–300 bp from the start codon ATG; the structure is compatible with the *Pi9* gene in the Nipponbare (NPB) reference genome (*Nbs2-Pi2/9-NPB*; DQ454158) (Zhou et al. 2007). On the other hand, *Pi9-169* has a 1-bp insertion (T) at nt. 247, which recovers the frameshift and the early stop codon of its parent TNG67 (Additional file 11: Fig. S2a). However, in the two types of CRISPR-edited plants, which included the plants with the knock-out of NBS-LRR2 in SA0169, and the other that recovered the early stop mutation of TNG67 to a read-through reading frame, suggested that the NBS-LRR2 might not be responsible for the blast resistance of SA0169 (Additional file 6: Table S6 and Additional file 11: Fig. S2b). These results suggested that other *R* candidates within the *Pi169-6*(*t*) are responsible for the blast resistance in SA0169. This issue requires a further study (Table 1).

**Table 1:** Candidate *R* genes identified in *Pi169-6*(*t*) and *Pi169-11*(*t*) of SA0169

Gene ID^a^	Location	Description
*Pi169-6*(*t*) genomic region		
LOC_Os06g17880^a^	Chr6:10,375,846–10,380,263	NBS-LRR disease resistance protein, putative, expressed
LOC_Os06g17920^a^	Chr6:10,405,659–10,415,295	NBS-LRR disease resistance protein, putative, expressed
*Pi169-11*(*t*) genomic region		
LOC_Os11g44970	Chr11:27,234,199–27,237,260	NBS-LRR disease resistance protein, putative, expressed
LOC_Os11g44990	Chr11:27,242,070–27,245,161	NB-ARC domain containing protein, expressed
LOC_Os11g45130	Chr11:27,318,942–27,324,163	Pollen signaling protein with adenylyl cyclase activity, putative, expressed
LOC_Os11g45620	Chr11:27,603,341–27,609,143	Rust-resistance protein Lr21, putative, expressed
LOC_Os11g45790	Chr11:27,703,761–27,707,310	NB-ARC domain containing protein, expressed
LOC_Os11g45840	Chr11:27,734,572–27,737,316	Expressed protein
LOC_Os11g45924	Chr11:27,789,618–27,791,943	WRKY41, expressed

^a^NBS-LRR1 in *Pi2*/*9* locus: LOC_Os06g17880; NBS-LRR4: LOC_Os06g17920

To understand the other *R* candidates in SA0169, the annotated *R* genes within the confidence region *Pi169-11*(*t*) (26.65 to 29.02 Mb; 2.37 Mb) were studied. The *Pi169-11*(*t*) genomic region is on the distal region of the long arm of chr. 11, which is co-localized with a rice *R* gene cluster including at least 28 *R* genes (Fig. 2b) (Wang et al. 2014). Our sequence analysis revealed that the *Pi169-11*(*t*) region includes 56 genes with 117 non-synonymous mutations, 11 of which are annotated as *R* genes. Of these 11 *R* genes, LOC_Os11g45980 is found to have a 2 bp insertion in SA0169, which causes a premature stop codon; three genes (LOC_Os11g45160, LOC_Os11g45780 and LOC_Os11g47447) have non-synonymous mutations locating in the non-functional domain regions that may not result in significant functional changes. The remaining 7 genes, carrying non-synonymous mutations on the functional domains, include LOC_Os11g45130 and LOC_Os11g45620, which encode a putative pollen signaling protein with adenylyl cyclase activity and rust-resistance protein Lr21, respectively, both of which are involved in the early stages of the interaction with the blast pathogen (Bagnaresi et al. 2012; Vijayan et al. 2013). They also include LOC_Os11g44970, LOC_Os11g44990, LOC_Os11g45790, LOC_Os11g45840 and LOC_Os11g45924, which are, respectively, a nucleotide-binding site leucine-rich repeat (NBS-LRR) protein, an NB-ARC domain containing protein, and a WRKY transcription factor, which belongs to the gene family involved in plant disease resistant network (McHale et al. 2006; Pandey and Somssich 2009; Ryu et al. 2006; van Ooijen et al. 2008). In summary, there are 7 genes identified as *R* candidates in *Pi169-11*(*t*) (Table 1 and Fig. 2c).

### Disease resistance breeding using *R* regions in SA0169

An objective of this study was to develop new blast and bacterial blight double resistance lines to increase the adaptability of the crop to environmental changes and to reduce the application of fungicides. To speed up the process of breeding, the genomic study of SA0169 and disease resistance breeding were conducted at the same time. The new disease resistant lines were established by using phenotype selection on disease responses, and combined with the marker-assisted backcrossing (MABC), which was assisted by the above genomic study of SA0169 (Additional file 12: Fig. S3). The recurrent parent was adopted by our previous bred broad-spectrum bacterial blight resistant line 966A1, which carries five (*Xa4, xa5, Xa7, xa13* and *Xa21*) bacterial blight resistant genes; and the blast resistance donor was SA0169, which harbors the two *R* regions (*Pi169-6*(*t*) and *Pi169-11*(*t*)). The gene pyramiding procedure and pedigree are shown in Additional file 12: Fig. S3.

**Fig. 3:**
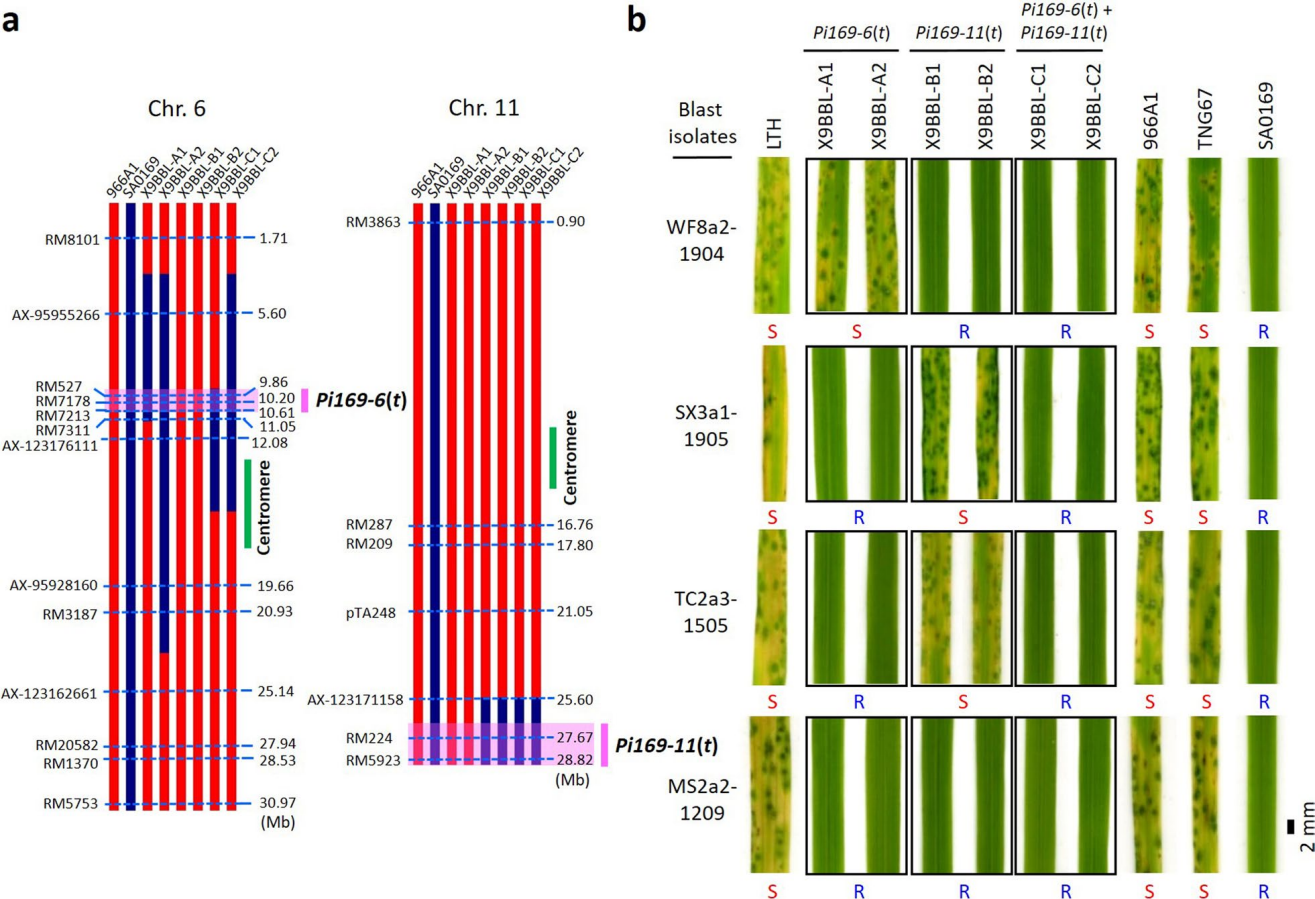
Validation of the blast resistance of the candidate regions using near-isogenic lines (NILs). **a** The genotyping of the NILs and the candidate regions of blast resistance of SA0169 were conducted using the SSR, functional (pTA248), and array (SNP) markers. Pink shaded regions: candidate blast resistant regions (*Pi169-6*(*t*), 9.63–10.79 Mb; *Pi169-11*(*t*), 26.65–29.02 Mb). **b** The investigation of blast responses. LTH and TNG67 are two blast susceptible lines; SA0169 is a blast resistant line; 966A1 is a blast susceptible line and the recurrent parent of the NILs. X9BBL-A1 and X9BBL-A2, 966A1 NILs carry *Pi169-6*(*t*); X9BBL-B1 and X9BBL-B2, 966A1 NILs carry *Pi169-11*(*t*); X9BBL-C1 and X9BBL-C2, 966A1 NILs carry double resistant regions (*Pi169-6*(*t*) and *Pi169-11*(*t*)). R, resistant response; S, susceptible response

Since the *R* genes mapping and disease resistance breeding started at the same time, the physical positions of the *R* regions in SA0169 (*Pi169-6*(*t*) and *Pi169-11*(*t*)) were not available until the linkage mapping and the QTL-seq mapping were completed. To ensure the selection of the progeny carrying the blast resistance and genotype in earlier generations (BC_1_ to BC_4_F_2_), the blast resistant offspring were selected by inoculating with mixed spore suspension, which was the mixture of 9 blast isolates from different counties in Taiwan (Additional file 7: Table S7). In theory, only the progeny harboring multiple *R* regions could meet the challenge of various isolates, and showed blast resistance. In addition to the phenotypic selection, the selection of blast resistant progeny was also confirmed by the blast resistance associated markers of the two *R* regions in later generations. The markers of *Pi169-6*(*t*) were developed from the linkage mapping and used in resistant progeny selection since the BC_2_ generation; the associated markers of *Pi169-11*(*t*) were developed from the QTL-seq study and utilized in the BC_4_F_2_ (Additional file 8: Table S8). To pyramid the bacterial blight resistance, the progeny carrying the homozygous bacterial blight resistant genotype (*Xa4, xa5, Xa7, xa13* and *Xa21*) were continuously selected in each generation (Additional file 8: Table S8). According to our previous experience in developing the bacterial blight resistant lines (XD9 and XD11), new lines with 4 or 5 *Xa* genes generally showed broad resistance against various Taiwanese bacterial blight pathogens. To ensure the bacterial blight resistance of the new double resistance lines, these new lines were challenged with 10 representative bacterial blight pathogens, which were collected from various counties by scientists at the Taiwan Agricultural Research Institute (Chen et al. 2015). All of the developed double resistant lines showed strong and broad spectrum resistance against all the tested bacterial blight pathogens (Additional file 13: Fig. S4). In addition to the blast and bacterial blight resistance genotypes, plants of good phenotypes and agronomic traits were selected in the breeding process. The genomic constitution of these lines was determined using SSR markers, functional markers, and the Axiom Rice Genotyping Array. In total, 33/751 PCR based markers (4.4%) and 3391/48460 array markers (7.0%) showed polymorphisms between the recurrent parent (966A1, a blast susceptible line) and the blast resistant donor SA0169, and the interpretable regions by these markers cover 96.1% of the genome. Finally, six lines of three types were obtained, including two lines carrying a single *Pi169-6*(*t*) (X9BBL-A1 and X9BBL-A2; also known as X9BBL-A lines), two lines carrying a single *Pi169-11*(*t*) (X9BBL-B1 and X9BBL-B2; known as X9BBL-B lines), and two lines carrying the double resistant NILs containing both *Pi169-6*(*t*) and *Pi169-11*(*t*) (X9BBL-C1 and X9BBL-C2; named as X9BBL-C lines) (Fig. 3a and Additional file 14: Fig. S5).

In order to control rice blast in Taiwan, plant pathologists at the Taiwan Agricultural Research Institute (TARI) have collected hundreds of blast isolates every year since 2010 and conducted the epidemiological study of blast pathogens in the field. To understand the blast resistance spectrum of these double resistant lines, these lines were inoculated with 16 representative blast isolates collected by TARI, which showed complementary virulence spectrum among each other and were selected by their pathotypes after the analyses of 1,552 isolates from 14 counties in Taiwan against 51 rice varieties/lines during 2014–2020 (Yi-Nian Chen et al., manuscript in preparation). The blast response assay indicated that the recurrent parent (966A1) and susceptible controls (TNG67 and LTH) were susceptible to all tested isolates, while SA0169 showed complete resistance. For the NILs, the X9BBL-A lines (*Pi169-6*(*t*)) displayed resistance against 8 blast isolates; X9BBL-B lines (*Pi169-11*(*t*)) showed resistance to 12 isolates; the X9BBL-C lines, which contain both *Pi169-6*(*t*) and *Pi169-11*(*t*), showed resistance to all 16 tested isolates (Table 2). Thus, 4 isolates were specifically recognized by *Pi169-6*(*t*), but not by *Pi169-11*(*t*); 8 isolates were recognized only by *Pi169-11*(*t*), but not by *Pi169-6*(*t*); 4 isolates (CT2a1(1)-1803, MS2a2-1209, EL1a2-1908, and CT2a1-1703) were recognized by both *Pi169-6* (*t*) and *Pi169-11*(*t*) (Table 2 and Fig. 3b). In summary, these results indicate that the resistance spectra of *Pi169-6* (*t*) and *Pi169-11*(*t*) are different but the two resistance regions are complementary, resulting in resistance to all tested blast isolates. Thus, the broad-spectrum, durable blast resistance of SA0169 is attributable to the complementary contributions from both *Pi169-6* (*t*) and *Pi169-11*(*t*).

**Table 2:** The blast resistance responses of tested lines containing genotypes of *Pi9*, *Pi169-6*(*t*), and/or *Pi169-11*(*t*)

	Genotype		*Pi169-6*(*t*)		*Pi169-11*(*t*)		*Pi169-6*(*t*) + *Pi169-11*(*t*)				
Blast isolates	Collected location	LTH^b^	X9BBL-A1	X9BBL-A2	X9BBL-B1	X9BBL-B2	X9BBL-C1	X9BBL-C2	966A1^b^	TNG67^b^	SA0169
EM1a1-1903	Emei, Hsinchu	S	R^a^	R	S	S	R	R	S	S	R
SX3a1-1905	Sanxing, Yilan	S	R	R	S	S	R	R	S	S	R
CT2a1(1)-1803	Caotun, Nantou	S	R	R	R	R	R	R	S	S	R
MN4a2-1804	Meinong, Kaohsiung	S	S	S	R	R	R	R	S	S	R
LY3a2-1805	Luye, Taitung	S	S	S	R	R	R	R	S	S	R
GS3a1-1903	Guanshan, Taitung	S	S	S	R	R	R	R	S	S	R
WD1a3-1803	Wandan, Pingtung	S	S	S	R	R	R	R	S	S	R
YUL4a3-1805	Yuli, Hualien	S	S	S	R	R	R	R	S	S	R
MS2a2-1209	Minxiong, Chiayi	S	R	R	R	R	R	R	S	S	R
TC2a3-1505	Toucheng, Yilan	S	R	R	S	S	R	R	S	S	R
WF8a2-1904	Wufeng, Taichung	S	S	S	R	R	R	R	S	S	R
EL1a2-1908	Erlin, Changhua	S	R	R	R	R	R	R	S	S	R
WF4a1-1404	Wufeng, Taichung	S	S	S	R	R	R	R	S	S	R
CT2a1-1703	Caotun, Nantou	S	R	R	R	R	R	R	S	S	R
MN2a3-1803	Meinong, Kaohsiung	S	S	S	R	R	R	R	S	S	R
LIY2a1-2008	Liouying, Tainan	S	R	R	S	S	R	R	S	S	R

^a^R, blast resistant response; S, susceptible response

^b^LTH: blast susceptible line Lijiangxintuanheigu. 966A1: recurrent parent of the NILs (X9BBL-A1, -A2, -B1, -B2, -C1, and -C2). TNG67: mutation parent of SA0169

## Discussion

The *R* genes conferring broad-spectrum resistance are highly valuable for improving rice blast resistance. The rice blast pathogen evolves rapidly in the field and a blast resistance cultivar usually breaks down within 3–5 years after large area monoculturing (Devi et al. 2015). Pyramiding of multiple *R* genes is generally considered an effective strategy to generate new varieties with broad-spectrum and durable resistance (Kalia and Rathour 2019). However, the resistance effects are not always simple accumulation of resistance spectra of target *R* genes; the interaction among the pyramided *R* genes may incur positive or negative effects on resistance. Thus, combinatory effects of *R* genes in the target genetic background should be assessed before breeding (Ning et al. 2020). In general, pyramiding multiple *R* genes require longer time and more efforts for breeders to generate new resistant varieties. Breeders prefer to utilize the varieties with broad-spectrum resistance conferred by a single *R* gene, because it substantially accelerates the breeding process (Li et al. 2019). Recently, a few *R* genes conferring broad-spectrum resistance (e.g. *Pi9*, *Pigm*, *Pi50*, *pi21*, *Pi57*, and *Ptr*) have been identified, most of which were derived from cultivars or wild species (Deng et al. 2017; Dong et al. 2017; Fukuoka et al. 2009; Qu et al. 2006; Zhao et al. 2018; Zhu et al. 2012). To discover more valuable *R* genes/alleles other than those from natural resources, this study focused on mapping the *R* genomic regions conferring the broad-spectrum resistance in an artificially induced mutant SA0169.

### Mapping the *R* genomic regions conferring blast resistance in SA0169

To understand the causal mutations in SA0169, the LTH×SA0169 F_2_ population and the linkage mapping method were first used to identify the *R* region(s) in SA0169. A single genomic region on Chr. 6 (*Pi169-6*(*t*)) was identified and this finding corresponds to the single-gene inheritance pattern of this population (Fig. 1a and Additional file 2: Table S2) (Wang et al. 2019a). However, the identified single *R* region may not be sufficient to confer the broad-spectrum resistance against the variable blast isolates in the field and fully explain the durable resistance of SA0169. Later, we noticed that the TNG67×SA0169 F_2_ population did not fit to 3:1, or 15:1 (Additional file 2: Table S2), suggesting that there were more un-identified *R* genes, cofactors, or repressors, which may be involved in the defense network of blast resistance in SA0169. The exact inherited model could not be determined until all the *R* genes within the identified *R* regions are functional annotated and validated. To efficiently map the *R* intervals, the QTL-seq strategy was applied to the TNG67×SA0169 F_2_ population. The principle behind QTL-seq is “bulk segregant analysis” (BSA) (Michelmore et al. 1991). In BSA, two bulks contrasting for the trait of interest (e.g. R/S bulks for disease resistance/susceptibility) from the segregating progenies (e.g., F_2_) is the foundation of mapping (Takagi et al. 2013; Tribhuvan et al. 2018). In this study, the preparation of R/S bulks was conducted by using a widely used plant-genetics method, called “progeny test”, to test the genotype of F_2_ plants by the phenotype segregation in their F_2:3_ offspring (Rosa 2013; Vengadessan et al. 2013; Zhu et al. 2007). The selection of F_2_ individuals for R/S bulks depended on the phenotypic data from the F_2:3_ progenies rather than the F_2_ plants. The application of this procedure was based on several special considerations regarding the blast resistance phenotyping: (1) Blast disease generally causes destructive damage of the susceptible seedlings, such that the high-quality and sufficient number of samples were hard to obtain for the following whole-genome resequencing. (2) Blast resistance response is highly dependent on the interactions among the host, pathogen and environment (Asibi et al. 2019); any perturbation of these factors may decrease the accuracy of phenotyping. Phenotyping a sufficient number of F_2:3_ plants in place of single F_2_ individual phenotyping can increase the accuracy of phenotyping. (3) By selecting the F_2_ individuals whose F_2:3_ progenies exhibited extreme phenotypes and no phenotypic segregation (Additional file 4: Table S4), the F_2_ plants in the presence or absence of multiple target *R* intervals with homozygous genotype could be identified and pooled as R- and S- bulks, respectively. The genomic constitution of the target regions in the two bulks should be highly distinct, which facilitates the identification and visualization of *R* regions shown in the Δ(SNP‐index) plot. In this study, the blast resistance reactions of ~ 2,700 F_2:3_ offspring derived from 155 F_2_ individuals were investigated. Two genomic regions (*Pi169-6*(*t*) and *Pi169-11*(*t*)) were successfully identified by this procedure and later their blast resistant capabilities were validated by using NILs (Table 2). The first QTL-seq study identified one *R* region *qPi-nor1*(t) from the rice cultivar Nortai by using 241 RILs, which were established from the F_7_ generation (Takagi et al. 2013). Our results indicated that QTL-seq incorporated with the progeny test required a shorter time and was feasible for mapping blast resistance in rice.

In this study two *R* regions were identified: *Pi169-6*(*t*) and *Pi169-11*(*t*). *Pi169-6*(*t*) was identified by the linkage mapping of the LTH×SA0169 F_2_ population and the QTL-seq mapping of the TNG67×SA0169 F_2_ population. On the other hand, the *Pi169-11*(*t*) was identified by using only the TNG67×SA0169 F_2_ population (Fig. 1). This approach is simpler because TNG67 is more similar to SA0169 than LTH and it suggested that genetic mapping is population dependent (LTH×SA0169 vs. TNG67×SA0169). A comparable example is a functional study of the *NLR* genes in the blast resistant cultivar Tetep (Wang et al. 2019b). The *NLRs* in Tetep were cloned and transformed into two different blast susceptible cultivars Shin2 and TP309, respectively. Two *NLRs* conferred resistance for the blast isolate S2007 when they were transformed into Shin2 but were susceptible when transformed into TP309; seven *NLRs* exhibited susceptibility in Shin2 but resistance in TP309 and two *NLRs* conferred resistance in both Shin2 and TP309 (Wang et al. 2019b). These results indicate that the blast resistance response of some *NLRs* may be altered under different genomic backgrounds. This phenomenon could be explained by the nature of complex defense networks in plants. In addition to *NLR* genes, many co-receptors and downstream signaling components are also involved in the complicated immune response of plants (Wu et al. 2018). Therefore, there might be some cofactors that interact with *Pi169-11*(*t*) are absent in the LTH genome, so that *Pi169-11*(*t*) was not identified in the LTH×SA0169 F_2_ population.

### Broad-spectrum blast resistance induced by sodium azide mutagenesis

The co-existence and complementarity of *Pi169-6*(*t*) and *Pi169-11*(*t*) is probably the main factor determining the broad-spectrum resistance of SA0169 against *P. oryzae*. It is supported by the fact that *Pi169-6*(*t*) and *Pi169-11*(*t*) have distinct resistance patterns but, in some cases, recognize the same isolates; the combination of their resistance patterns is equal to that of SA0169, which shows complete resistance to all isolates tested (Table 2). These observations explain why SA0169, which carries both *Pi169-6*(*t*) and *Pi169-11*(*t*), has maintained the durable blast resistance for ~ 20 years. Broad-spectrum blast resistance controlled by multiple genomic regions was also reported in previous studies (Chaipanya et al. 2017; Zhang et al. 2015). One example is a Thai rice variety Jao Hom Nin (JHN), which showed broad-spectrum resistance against the Thai and Philippine blast isolates (Chaipanya et al. 2017). Its high level of resistance can be attributed to the additive contributions of two *R* genes *Pish-J* and *Pi7-J*, which are on chr. 1 and 11, respectively (Chaipanya et al. 2017). Two other comparable examples are the durable blast resistant germplasms Tetep and Gumei 2, which harbor abundant functional *R* genes that provided the redundancy in the ability to recognize fast-evolving blast pathogen in the field and promoted the durable resistance of rice varieties (Zhang et al. 2015). The above data showed that sodium azide can generate genome-wide mutations that alter the functions of *R* genes in the genome (Wang et al. 2019a). Given that other *R* genes besides *Pi169-6*(*t*) and *Pi169-11*(*t*) may also be modified by sodium azide mutagenesis, the functional effect of genetic variants in SA0169 was predicted. Our analysis using the SnpEff tool indicated that the sodium azide induced mutations may incur moderate and high impacts on ~ 80 *R* genes throughout the SA0169 genome (Additional file 9: Table S9), suggesting that more functional *R* genes might be associated with the durable resistance in SA0169.

### Application of the SA0169 in resistance breeding

In this study, the bacterial blight (BB) resistant *Xa* genes were on the recurrent parent 966A1 genome, while the blast (BL) resistant region *Pi169-11*(*t*) was from SA0169. According to the map position, both *Xa4* and *Pi169-11*(*t*) are near the telomeric end of the long arm of chromosome 11 (Xu et al. 2008). Since the positions of *Xa4* and *Pi169-11*(*t*) might overlap, recombination between the *Xa4* and the *R* genes in *Pi169-11*(*t*) should rarely occur. In this study, we did not obtain the progeny carrying both *Xa4* and *Pi169-11*(*t*), which was likely due to the limited population size during the screening process. However, this should not influence the achievement of the objectives in this breeding program. Our previous study regarding the resistance responses of the five *Xa* genes (*Xa4, xa5, Xa7, xa13,* and *Xa21*) had shown that the individuals carrying 4 or 5 *Xa* genes can provide sufficient broad spectrum resistance against the various bacterial blight pathogens in Taiwan. The resistance response study in this study also showed that the X9BBL lines have good resistance against the tested BB pathogens. For breeding new varieties with better disease resistance, the three new NILs established in this study (X9BBL-A, X9BBL-B, and X9BBL-C lines) provided different blast resistance spectra and may be adopted in other breeding programs. They exhibit both blast and bacterial blight resistances and good agronomic traits, so they are ready to be released to farmers.

## Conclusions

**Fig. 4:**
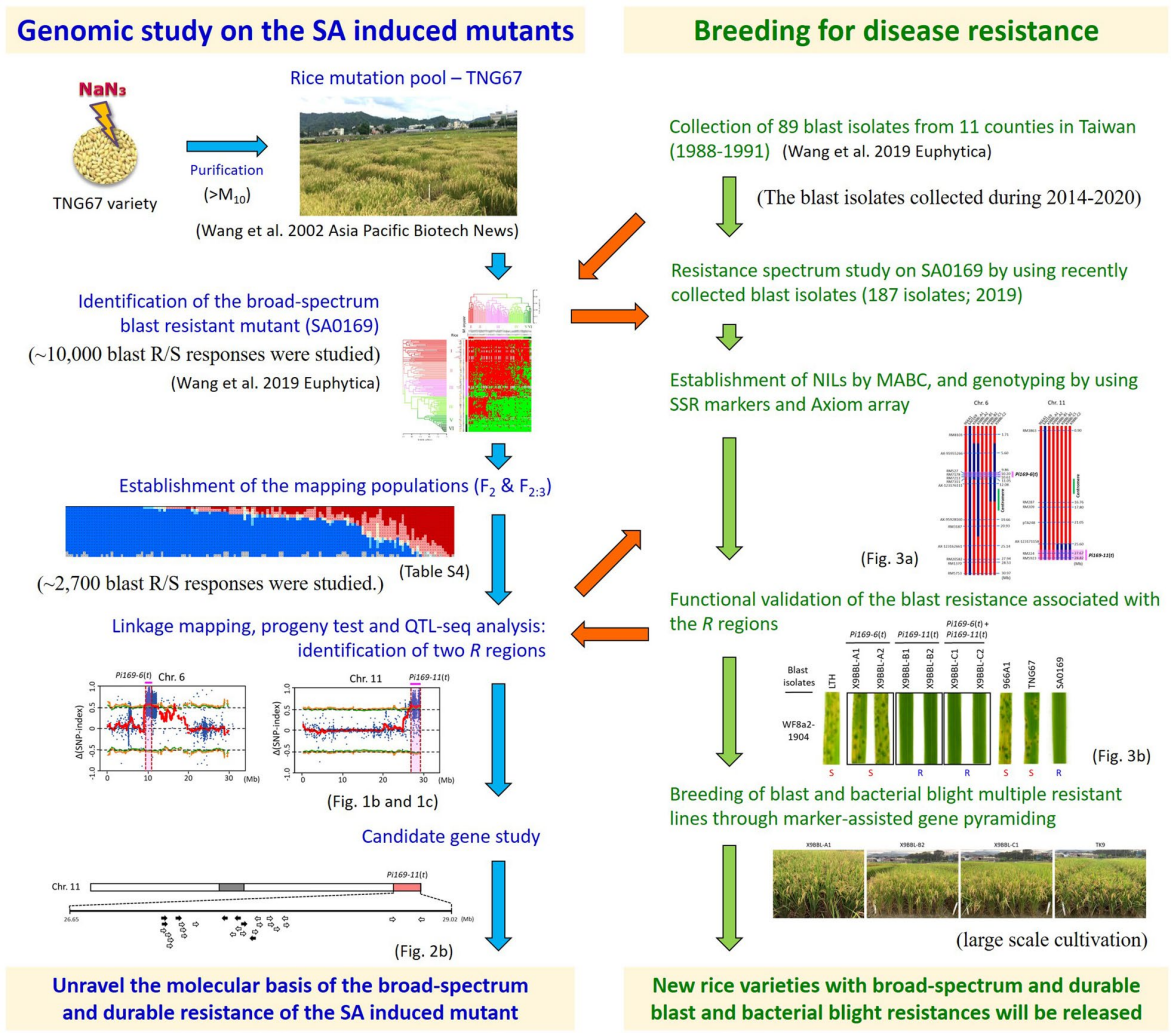
Overview of the genomic study and disease resistance breeding using the blast resistant mutant SA0169. Left panel is the flowchart of genomic study in SA0169. The procedure of combining the progeny test and QTL-seq accelerated the mapping process of the blast resistance associated regions in SA0169. Right panel is the steps of breeding resistant lines, which included the selection of a bacterial blight resistant line as the recurrent parent, and the establishment of NILs through MABC with the mapping results. The developed lines can be used as disease resistance donors for BL and BB, and are ready to be released to farmers. This study provides an example for the combination of a genomic study on a SA induced mutant and breeding for disease resistance. BL, blast; BB, bacterial blight; SA, sodium azide; NILs, near isogenic lines; MABC, marker-assisted backcrossing

An overview of the experimental designs in this study is summarized in Fig. 4. In conclusion, by using linkage mapping, progeny test, and QTL-seq strategy, two genomic regions (*Pi169-6*(*t*) and *Pi169-11*(*t*)) conferring the blast resistance in a rice sodium azide induced mutant SA0169 was identified. The combination of the resistance spectra of these two regions explains why SA0169 has displayed broad-spectrum and durable blast resistance for ~ 20 years. Our results improve the understanding of the genetic basis of the broad-spectrum blast resistance induced by sodium azide and provide a basis for breeding new rice varieties with durable blast resistance.

## Materials and methods

### Rice materials

SA0169 is a broad-spectrum blast resistant mutant line identified from the mutant pool of Taiwan *japonica* cultivar Tainung 67 (TNG67) (Wang et al. 2002), which was an extensively planted variety during 1979–1998 in Taiwan. TNG67 is high yield, lodging resistant, but highly susceptible to blast pathogen (Buu and Huang 1983; Kuo 1981; Wang et al. 2019a). 966A1 is the sister line of Xinda 9 (XD9; also known as CS9) (Wang et. al., 2018, Plant Variety Rights (PVR) pending), which was developed through marker-assisted backcrossing (MABC) by the introduction of five bacterial blight (BB) resistant genes (*Xa4, xa5, Xa7, xa13,* and *Xa21*) of IRBB66 into the elite variety Taikeng 9 (TK9), that was famous for its superior grain quality in Taiwan (Bai 2016). The near isogenic lines (NILs) of X9BBL (Xinda 9 with both Bacterial blight and BLast double resistance) applied in this study were developed by the introduction of the blast resistance of SA0169 (containing *Pi169-6*(*t*) and *Pi169-11*(*t*)) into the bacterial blight resistant line 966A1 also through the MABC strategy. X9BBL-A lines (X9BBL-A1 and X9BBL-A2) carry five BB resistant genes (*Xa4, xa5, Xa7, xa13,* and *Xa21*), and blast resistant gene(s) *Pi169-6*(*t*). X9BBL-B lines (X9BBL-B1 and X9BBL-B2) have four BB resistant genes (*xa5, Xa7, xa13,* and *Xa21*) and blast resistant gene(s) *Pi169-11*(*t*); X9BBL-C lines (X9BBL-C1 and X9BBL-C2) carry four BB resistant genes (*xa5, Xa7, xa13,* and *Xa21*), and two blast resistant genes *Pi169-6*(*t*) and *Pi169-11*(*t*) (Chiu 2020).

### Evaluation of blast and bacterial blight resistance

Rice blast pathogen (*Pyricularia oryzae*; syn. *Magnaporthe oryzae*) inoculum preparation followed the methods described by Chen et al. (2021). For inoculation, the rice seedlings (four leaf stage) were inoculated with conidial suspensions at a density of 1×10^5^ conidia/ml with 0.05% (v/v) Tween 80. After inoculation, plants were incubated in dew chamber at 26℃ with 95–100% relative humidity for 7 days. The disease reactions were classified by a scale of six scores from 0 (R) to 5 (S); scores 0 to 3 are classified as resistant response (R) and scores 4 and 5 are classified as susceptible response (S) (Bonman et al. 1986; Chen et al. 2013). The resistance frequency (RF) to the blast isolates were the ratio of the number of incompatible isolates to the total isolates used ×100% (Wang et al. 2019a; Wu et al. 2015). Moreover, the evaluation of the bacterial blight resistance was based on the method described by Tseng et al. (2015). In short, at the tilling stage, we inoculate the pathogens (*Xanthomonas campestris* pv. *oryzae*) into the plants by using leaf clipping method (Kauffman et al. 1973). The length of the lesions of the lines was measured and photographed at the 28–30 days after inoculation.

### Linkage mapping

The genomic DNA extraction of F_2_ individuals and two parents (TNG67 and SA0169) and genotyping by using SSR markers followed the descriptions of Tseng et al. (Tseng et al. 2015). The linkage mapping was conducted using the R software with add-on package R/qtl (version: 1.46–2) (Broman et al. 2003). The simple interval mapping (SIM) model was employed and genetic distances were estimated by using the Kosambi map function. Significant logarithm of odds (LOD) threshold (*p* < 0.05) was calculated through 1,000 genome-wide permutations. The Bayesian credible region-method was applied to calculate the 95% confidence intervals.

### QTL-seq method

Whole-genome sequencing was conducted in combination with Illumina MiSeq, and HiSeq2500 sequencing by the High-Throughput Sequencing Core of Academia Sinica, Taiwan. The obtained reads were then subjected to pre-processing and elimination of low-quality bases to remove poor-quality reads. QTL-seq analysis follow the methods described by Takagi et al. (2013) and the instruction (ver. 2.1.2) shown in the GitHub website (https://github.com/YuSugihara/QTL-seq). In brief, the raw reads were trimmed using Trimmomatic (ver. 0.36) with default parameters (Bolger et al. 2014). The trimmed reads of TNG67 (34,276,144 reads) and two bulks with extreme opposite traits (R *vs*. S responses; 153,733,728 and 156,898,612 reads, respectively) were aligned and mapped onto the rice genome (Os-Nipponbare-Reference-IRGSP-1.0) by using BWA-MEM (Li and Durbin 2009). The quality of alignment was evaluated by using the Qualimap2 software (ver. 2.2.1) (Okonechnikov et al. 2015). The conversion and sorting of the alignment files, and SNP calling were conducted by SAMtools (ver. 1.8) and BCFtools (ver.1.8), respectively (Li 2011; Li et al. 2009). The calculation of the SNP index and Δ(SNP‐index) ((SNP‐index of R‐bulk) – (SNP‐index of S‐bulk)) followed the methods described by Takagi et al. (2013). The sliding window analysis was applied to the SNP‐index plots with 1 Mb window size and 10 kb increment.

### Cloning of the *Pi9* gene

To verify the gene structure of *R* region (*Pi169-6*(*t*)) in SA0169 identified from the linkage mapping, progeny test, and QTL-seq, the *Pi9-169* and the *Pi9* of TNG67 (mutation parent) and SA169 were cloned and sequenced by the Sanger sequencing method: The total RNA extracted from 10-day-old TNG67 and SA0169 seedlings were used for cDNA synthesis by reverse transcription using RevertAid RT Reverse Transcription Kit (Thermo Fisher Scientific). The primer set (Nbs2/Pi9-F, 5′–TAACTAGTATGGCGGAGACGGTGCTGAG and Nbs2/Pi9-R, 5′-TAACTAGTTCAGCCAGCTTGAGCTGTGC) was designed to amplify the *Pi9* gene by PCR using Phusion High-Fidelity DNA Polymerase (Thermo Fisher Scientific). The amplified fragments were cloned into the pGEM-T easy vector (Promega) for sequence analysis and comparisons. The sequences have been confirmed by at least two repeats of the PCR products.

### High-density array genotyping and sequence analysis

The Axiom Rice Genotyping Array (Thermo Fisher Scientific Inc.) and the Axiom Analysis Suite Software (version 4.0.1.9) was employed to identify the genetic variants and genotypes of the NILs. Variant annotation and effect prediction was conducted by the SnpEff tool (ver. 4.3) (Cingolani et al. 2012). The annotated rice *R* genes were surveyed from the OryGenesDB database (https://orygenesdb.cirad.fr/) and previous reports (Droc et al. 2009; Li et al. 2019; Luo et al. 2012). The rice reference genomic sequences and annotation were adopted from the version of Os-Nipponbare-Reference-IRGSP-1.0 pseudomolecules and MSU Rice Genome Annotation Project Release 7 (http://rice.plantbiology.msu.edu/index.shtml) (Kawahara et al. 2013). The genotype data of 4,726 accessions of cultivated rice is adopted from the Rice Variation Map v2.0 database (RiceVarMap 2.0) (http://ricevarmap.ncpgr.cn/) (Zhao et al. 2015). Sequence alignment, annotation, and visualization of biological objectives were conducted by Bioedit, Unipro UGENE, and R package karyoplote R (Gel and Serra 2017; Hall 1999; Rose et al. 2019). Prediction of sequence translation was conducted by the tool at the ExPASy (https://web.expasy.org/translate/) (Artimo et al. 2012), and the conserved domains of the protein was predicted by using the CDD software (https://www.ncbi.nlm.nih.gov/Structure/cdd/wrpsb.cgi/) (Lu et al. 2020), and the SMART web resource (http://smart.embl-heidelberg.de/) (Letunic et al. 2021).

### Resistant gene validation by gene editing

To confirm the blast resistance of the *R* genes identified in SA0169, the gene editing experiment was conducted using the CRISPR/Cas9 technique. The procedure includes: selection of genomic targets, design of sgRNA (single guide RNA), construction of sgRNA-Cas9 expression cassette, cloning into a binary vector, delivering the gene construct into target plants using *Agrobacterium*-mediated transformation, regeneration of transgenic plants, and screening for gene editing events by PCR according to the methods described by Hsieh et al. (2021). For the analysis of CRISPR-edited plants, the modifications of the target site sequence were first analyzed at the T_0_ generation, and confirmed at the next generation. At least three independent homozygous transgenic T_1_ or T_2_ lines were obtained for each construct. Ten or more transgenic plants of each independent lines were used for blast resistance response study. The results of gene-edited plants are shown in Additional file 6: Table S6 and Additional file 11: Figure S2b.

## Supplementary Information

**Additional file 1: Table S1.** Response of 22 rice varieties/lines to 187 blast isolates challenge (2019)

**Additional file 2: Table S2.** Segregation of rice blast resistance in the F_2_ populations

**Additional file 3: Table S3.** Position and effect of the blast resistance-associated region identified from the LTH×SA0169 F_2_ population

**Additional file 4: Table S4.** Blast resistance response of F_2:3_ offspring derived from 155 F_2_ individuals of the TNG67×SA0169 cross

**Additional file 5: Table S5.** Summary of sequencing depth and coverage

**Additional file 6: Table S6.** List of the gene-edited plants of the NBS-LRR2 in TNG67 and SA0169 through the CRISPR/Cas9 technique

**Additional file 7: Table S7.** The origin and composition of mixed rice blast isolates

**Additional file 8: Table S8.** Molecular markers of the *Xa* genes, *Pi169-6*(*t*), and *Pi169-11*(*t*) for marker-assisted backcrossing

**Additional file 9: Table S9.** Candidate *R* genes in SA0169

**Additional file 10: Figure S1.** Two genomic regions responsible for the blast resistance in SA0169 were identified

**Additional file 11: Figure S2.** Blast resistance responses of the gene-edited plants obtained by the CRISPR/Cas9 technique

**Additional file 12: Figure S3.** The breeding scheme of the lines with blast and bacterial blight double resistance

**Additional file 13: Figure S4.** Disease resistance study against rice bacterial blight (BB) pathogen *Xanthomonas oryzae* pv. *oryzae* (*Xoo*) XF-89b strain

**Additional file 14: Figure S5.** Plant morphology of the newly developed blast and bacterial blight double resistant lines

## Data Availability

The data sets supporting the results of this article are included within the article and its supporting files.

## Abbreviations

CC: Coiled-coil domain
CRISPR: Clustered regularly interspaced short palindromic repeats
INDELs: Insertion/deletion
LOD: Logarithm of the odds
LRR: Leucine-rich repeat domains
MABC: Marker-assisted back crossing
MAS: Marker-assisted selection
NBS: Nucleotide-binding site domains
NILs: Near isogenic lines
NLR: Nucleotide-binding site leucine-rich repeat
NPB: Nipponbare
PCR: Polymerase chain reaction
QTLs: Quantitative trait locus
SA: Sodium azide
SNPs: Single nucleotide polymorphism
SSR: Simple sequence repeats
