# Supplementary material for: Two genomic regions of a sodium azide induced rice mutant confer broad-spectrum and durable resistance to blast disease
Source: Rice (N Y). 2022 Jan 10;15:2. doi: 10.1186/s12284-021-00547-z (PMC8748607; doi:10.1186/s12284-021-00547-z)
Supplement: Supplementary file 1 — Additional file 1: Table S1. Response of 22 rice varieties/lines to 187 blast isolates challenge (2019) [file 12284_2021_547_MOESM1_ESM.docx]

| **Table S1** Response of 22 rice varieties/lines to 187 blast isolates challenge (2019) | | | |
| --- | --- | --- | --- |
| **Variety/Line** | **R^a^** | **S^b^** | **Resistance frequency (%)** |
| SA0169 (Sodium azide induced mutant) | 187 | 0 | 100.0 |
| Taichung Sen 10 (TCS10) | 187 | 0 | 100.0 |
| Taikeng Glutinous 1 (TKG1) | 187 | 0 | 100.0 |
| Tainung 84 (TNG84) | 186 | 1 | 99.5 |
| Tainung 79 (TNG79) | 186 | 1 | 99.5 |
| Taitung 30 (TT30) | 184 | 3 | 98.4 |
| Taichung 192 (TC192) | 176 | 11 | 94.1 |
| Taichung Sen 17 (TCS17) | 168 | 19 | 89.8 |
| Tainung Waxy 73 (TNW73) | 158 | 29 | 84.5 |
| Taikeng 8 (TK8) | 151 | 36 | 80.7 |
| Tainung 77 (TNG77) | 132 | 55 | 70.6 |
| Kaohsiung 147 (KH147) | 128 | 59 | 68.4 |
| Taikeng 2 (TK2) | 98 | 89 | 52.4 |
| Kaohsiung 139 (KH139) | 89 | 98 | 47.6 |
| Taikeng 9 (TK9) | 79 | 108 | 42.2 |
| Kaohsiung 145 (KH145) | 73 | 114 | 39.0 |
| Tainung 71 (TNG71) | 64 | 123 | 34.2 |
| Taikeng 16 (TK16) | 63 | 124 | 33.7 |
| Hualien 21 (HL21) | 60 | 127 | 32.1 |
| Tainan 11(TN11) | 51 | 136 | 27.3 |
| Taoyuan 3 (TY3) | 39 | 148 | 20.9 |
| Taikeng 14 (TK14) | 31 | 156 | 16.6 |
| ^a^Number of varieties/lines showing resistant response (R) | | | |
| ^b^Number of varieties/lines showing susceptible response (S) | | | |
