# Supplementary material for: Two genomic regions of a sodium azide induced rice mutant confer broad-spectrum and durable resistance to blast disease
Source: Rice (N Y). 2022 Jan 10;15:2. doi: 10.1186/s12284-021-00547-z (PMC8748607; doi:10.1186/s12284-021-00547-z)
Supplement: Supplementary file 2 — Additional file 2: Table S2. Segregation of rice blast resistance in the F2 populations [file 12284_2021_547_MOESM2_ESM.docx]

| **Table S2** Segregation of rice blast resistance in the F_2_ populations | | | | | | | |  |  |  |
| --- | --- | --- | --- | --- | --- | --- | --- | --- | --- | --- |
| **Population** | **R^a^** | **S^a^** | **Total No.** | **Tested ratio** | ***χ*^2 b^** | ***P* value** | **Blast isolate** |  |  |  |
| LTH🞨SA0169 F_2_^c^ | 93 | 29 | 122 | 3:1 | 0.10 | 0.75 | MS2a2-1209 |  |  |  |
| TNG67🞨SA0169 F_2_ | 836 | 132 | 968 | 3:1 | 66.67 | <1🞨10^-5^ | MS2a2-1209 |  |  |  |
| TNG67🞨SA0169 F_2_ | 836 | 132 | 968 | 15:1 | 90.13 | <1🞨10^-5^ | MS2a2-1209 |  |  |  |
| ^a^R, resistant (score 0-3); S, susceptible (score 4-5)  ^b^*χ*^2^ _(0.05, 1)_ =3.84  ^c^Data from our previous report (Wang et al., 2019a) | | | | | | | |  |  |  |
