# Supplementary material for: Two genomic regions of a sodium azide induced rice mutant confer broad-spectrum and durable resistance to blast disease
Source: Rice (N Y). 2022 Jan 10;15:2. doi: 10.1186/s12284-021-00547-z (PMC8748607; doi:10.1186/s12284-021-00547-z)
Supplement: Supplementary file 3 — Additional file 3: Table S3. Position and effect of the blast resistance-associated region identified from the LTH×SA0169 F2 population [file 12284_2021_547_MOESM3_ESM.docx]

| **Table S3** Position and effect of the blast resistance-associated region identified from the LTH🞨SA0169 F_2_ population | | | | | |
| --- | --- | --- | --- | --- | --- |
| **Chr.** | **Blast resistance associated region**  **cM (CI)^a^** | **LOD score at peak** | **Var. (%)^b^** | **Add.^c^** | **Dom.^d^** |
| 6 | 55 (52-65) | 12.3 | 45.5 | -1.30 | -1.98 |
| ^a^CI, confidence interval  ^b^Percentage of variance explained by QTL  ^c^Add., additive effect  ^d^Dom. dominance effect | | | | | |
