# Supplementary material for: Two genomic regions of a sodium azide induced rice mutant confer broad-spectrum and durable resistance to blast disease
Source: Rice (N Y). 2022 Jan 10;15:2. doi: 10.1186/s12284-021-00547-z (PMC8748607; doi:10.1186/s12284-021-00547-z)
Supplement: Supplementary file 5 — Additional file 5: Table S5. Summary of sequencing depth and coverage [file 12284_2021_547_MOESM5_ESM.docx]

**Table S5** Summary of sequencing depth and coverage

| **DNA materials** | **Raw reads** | **Read length (bp)** | **Sequence depth**  **(X)^a^** | **Remained pairs**  **after trimming^b^** | **Mapped reads^c^** | **Genome coverage^c^** |
| --- | --- | --- | --- | --- | --- | --- |
| TNG67 | 39,469,402 | 300 | 31.78 | 17,138,072 | 32,516,155 | 96.95% |
| SA0169 | 42,695,592 | 300 | 34.38 | 18,163,922 | 35,566,825 | 97.65% |
| R-bulk | 165,330,076 | 150 | 66.78 | 76,866,864 | 149,512,789 | 98.77% |
| S-bulk | 167,776,976 | 150 | 67.78 | 78,449,306 | 153,478,620 | 98.76% |

^a^Sequenced depth = input reads * read length / Nipponbare genome size (373,795,655 bp)

^b^The raw reads were trimmed by using Trimmomatic (ver. 0.36) with default parameters (Bolger et al., 2014)

^c^The quality of alignment were evaluated by using the Qualimap2 software (ver. 2.2.1) (Okonechnikov et al., 2015)
