# Supplementary material for: Two genomic regions of a sodium azide induced rice mutant confer broad-spectrum and durable resistance to blast disease
Source: Rice (N Y). 2022 Jan 10;15:2. doi: 10.1186/s12284-021-00547-z (PMC8748607; doi:10.1186/s12284-021-00547-z)
Supplement: Supplementary file 6 — Additional file 6: Table S6. List of the gene-edited plants of the NBS-LRR2 in TNG67 and SA0169 through the CRISPR/Cas9 technique [file 12284_2021_547_MOESM6_ESM.docx]

**Table S6** List of the gene-edited plants of the NBS-LRR2 in TNG67 and SA0169 through CRISPR technology

| **Name of plants** | **Variety/line**  **(Host)** | **Plant No.** | **Genera-**  **tion** | **Construct/Gene** | **Edited Event** | **Number of plant** | **No. of homozygous edited plants** | | **Anticipated Result** | **Experimental**  **Result** |
| --- | --- | --- | --- | --- | --- | --- | --- | --- | --- | --- |
|  |  |  |  |  |  |  | **w/o**  **T-DNA** | **w/**  **T-DNA** |  |  |
| TG-1 | TNG67 | 15-1#5 | T_1_ | CRP-Nbs2 (read-through) 15-1 | +1 | 12 | 1 | 2 | S 🡢 R | S |
| TG-2 | TNG67 | 17-1#8 | T_1_ | CRP-Nbs2 (read-through) 17-1 | -2 | 12 | 3 | 0 | S 🡢 R | S |
| TG-3 | TNG67 | 11-1#10-10 | T_2_ | CRP-Nbs2 (read-through) 11-1#10 | -2 | 25 | 0 | 2 | S 🡢 R | S |
| TG-4 | SA0169 | 2-2#20-1 | T_2_ | CRP-Nbs2 (knockout) 2-2#20 | +1 | 12 | 0 | 2 | R 🡢 S | R |
| TG-5 | SA0169 | 4-1#16-1 | T_2_ | CRP-Nbs2 (knockout) 4-1#16 | -28 | 12 | 2 | 0 | R 🡢 S | R |
| TG-6 | SA0169 | 6-1#3-4 | T_2_ | CRP-Nbs2 (knockout) 6-1#3 | +1 | 12 | 2 | 0 | R 🡢 S | R |

TG1, TG2, and TG3: The CRISPR-edited plants which recovered the early stop mutation of NBS-LRR2 in TNG67 as read-through reading frame; TG4, TG5, and TG6: The gene-edited plants with knock-out of NBS-LRR2 in SA0169. CRISPR: Clustered Regularly Interspaced Short Palindromic Repeats; S, susceptible response; R, resistant response. Experimental results were conducted by inoculation with blast isolate EM1a1-1903.
