# Supplementary material for: Two genomic regions of a sodium azide induced rice mutant confer broad-spectrum and durable resistance to blast disease
Source: Rice (N Y). 2022 Jan 10;15:2. doi: 10.1186/s12284-021-00547-z (PMC8748607; doi:10.1186/s12284-021-00547-z)
Supplement: Supplementary file 7 — Additional file 7: Table S7. The origin and composition of mixed rice blast isolates [file 12284_2021_547_MOESM7_ESM.docx]

**Table S7** The origin and composition of mixed rice blast isolates

| **Blast isolates** | **Collected location** |
| --- | --- |
| XS1a1(1)-1503 | Xiushui, Changhua |
| NT1a3-1503 | Nantou City, Nantou |
| JY1a21-1504 | Chiayi Agricultural Experiment Branch, Chiayi |
| LG1a3-1504 | Ligang, Pingtung |
| TC2a2-1505 | Toucheng, Yilan |
| TC2a3-1505 | Toucheng, Yilan |
| HW1a3-1503 | Huwei, Yunlin |
| TD3a2-1505 | Taitung City, Taitung |
| JX2a2-1505 | Jiaoxi, Yilan |
