# Supplementary material for: Two genomic regions of a sodium azide induced rice mutant confer broad-spectrum and durable resistance to blast disease
Source: Rice (N Y). 2022 Jan 10;15:2. doi: 10.1186/s12284-021-00547-z (PMC8748607; doi:10.1186/s12284-021-00547-z)
Supplement: Supplementary file 8 — Additional file 8: Table S8. Molecular markers of the Xa genes, Pi169-6(t), and Pi169-11(t) for marker-assisted backcrossing [file 12284_2021_547_MOESM8_ESM.docx]

| **Table S8** Molecular markers of the *Xa* genes, *Pi169-6*(*t*), and *Pi169-11*(*t*) for marker-assisted backcrossing | | | | | |
| --- | --- | --- | --- | --- | --- |
| **Gene** | **Chr.** | **Marker** | **Forward (5'→3') primer** | **Reverse (5'→3') primer** | **Type** |
| *Xa4* and *Pi169-11*(*t*) | 11 | RM224 | ATCGATCGATCTTCACGAGG | TGCTATAAAAGGCATTCGGG | SSR |
| *Xa4* and *Pi169-11*(*t*) | 11 | RM5923 | ATAGTTCGGGGGGTAATTCG | GTCGATCGAGATAGTTGGGG | SSR |
| *xa5* | 5 | RM601 | CCGGGGGTGTTGGGCTTAT | TCACTGGCTCTACTTCCGCTTCAC | SSR |
| *xa5* | 5 | RM611 | CAACAAGATGGCCTCTTACC | TACAAACAAACAGCTTGTGC | SSR |
| *Xa7* | 6 | RM20582 | AGAGCGTCGTCCTTCACCATCC | GGCCAATACGACGATACATTACACG | SSR |
| *Xa7* | 6 | RM3138 | TTGACAAGAGATCAAGGCGG | GTGAATGTTGAGCTGCATGG | SSR |
| *xa13* | 8 | RM447 | CCCTTGTGCTGTCTCCTCTC | ACGGGCTTCTTCTCCTTCTC | SSR |
| *xa13* | 8 | RM3761 | CCTCAACAATAGCACCACCC | CTGCAAGTCTGCAAGCACAG | SSR |
| *Xa21* | 11 | pTA248 | AGCCGCGGAAGGGTGGTTCCCGGA | AGACGCGGTAATCGAAAGATGAAA | STS |
| *Pi169-6*(*t*) | 6 | RM7311 | AGTGGTCGTTGAACTCGGAG | TCGTGGCGCCTTTAATCTC | SSR |
| *Pi169-6*(*t*) | 6 | RM7178 | TAACCTTCACAGCGAACGTG | CCGTGAGATGGGCTACCTAC | SSR |
| *Pi169-6*(*t*) | 6 | RM7213 | CTATAGCCAGCGACGAGGAC | CTGCACCCATCTCTCTCTCC | SSR |
| Chr., chromosome; SSR, Simple Sequence Repeats; STS, Sequence-Tagged Site | | | | | |
