# Supplementary material for: Two genomic regions of a sodium azide induced rice mutant confer broad-spectrum and durable resistance to blast disease
Source: Rice (N Y). 2022 Jan 10;15:2. doi: 10.1186/s12284-021-00547-z (PMC8748607; doi:10.1186/s12284-021-00547-z)
Supplement: Supplementary file 10 — Additional file 10: Figure S1. Two genomic regions responsible for the blast resistance in SA0169 were identified [file 12284_2021_547_MOESM10_ESM.docx]

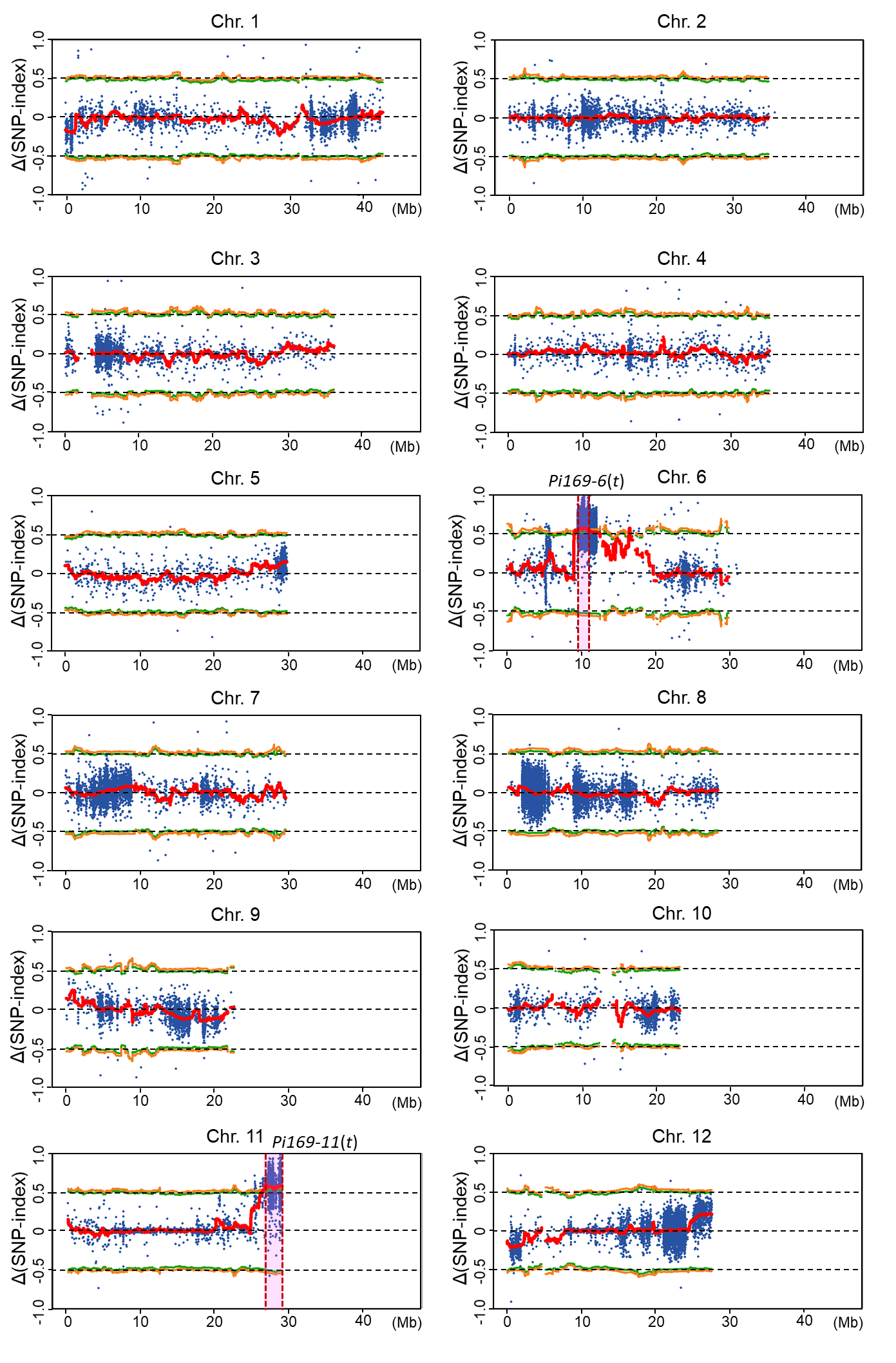


**Fig. S1** Two genomic regions responsible for the blast resistance in the SA0169 mutant were identified. The QTL-seq in combining with bulk segregant analysis (BSA) was applied to the F_2_ population of TNG67/SA0169 for SNP identification. Δ(SNP‐index) plot with statistical confidence intervals under the null hypothesis of no QTL s (green lines, *P* < 0.05; orange lines, *P* < 0.01). Blue dots, ∆ (SNP­‐index); Red lines, the average Δ(SNP‐index) in a 1 Mb interval using a 10 kb sliding window. Pink shaded regions on chromosome 6 and chromosome 11, the blast resistance candidate regions *Pi169-6*(*t*) and *Pi169-11*(*t*), respectively.
