# Supplementary material for: Two genomic regions of a sodium azide induced rice mutant confer broad-spectrum and durable resistance to blast disease
Source: Rice (N Y). 2022 Jan 10;15:2. doi: 10.1186/s12284-021-00547-z (PMC8748607; doi:10.1186/s12284-021-00547-z)
Supplement: Supplementary file 11 — Additional file 11: Figure S2. Blast resistance responses of the gene-edited plants obtained by the CRISPR/Cas9 technique [file 12284_2021_547_MOESM11_ESM.docx]

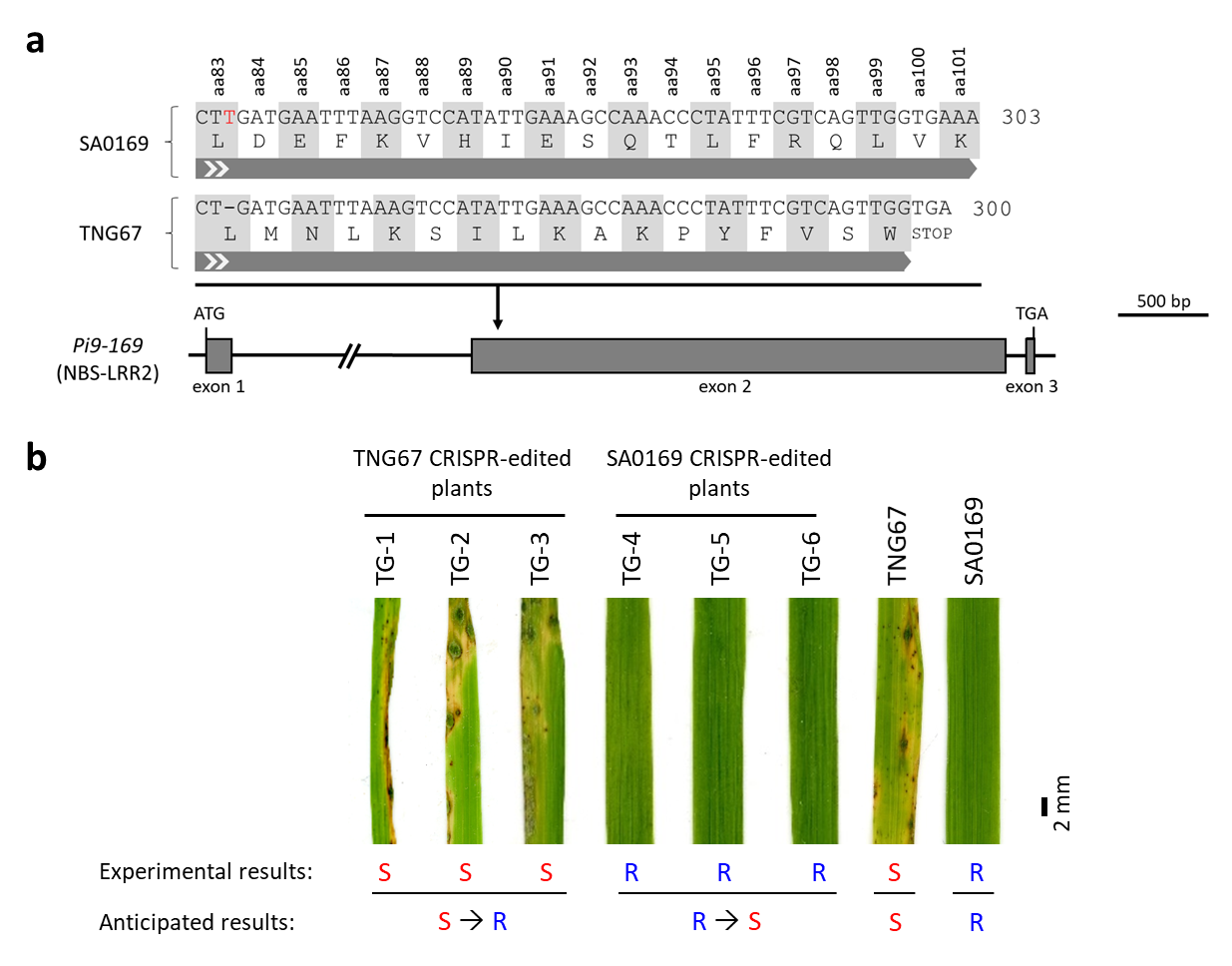


**Fig. S2** Blast resistance responses of the gene-edited plants through CRISPR technology. (**a**) Gene structure of *Pi9-169* (NBS-LRR2) in SA0169. Top row, nucleotide sequence; Middle row, amino acids sequence; Bottom row, gene ORF. The upper grey bar represents the translation in SA0169 which reads through and produces a complete protein due to a single T insertional mutation. The bottom grey bar shows an early stop in translation and produces a very short polypeptide that might have no function in TNG67. There is a single “T” insertion (red) at nt. 247 in SA0169, which recovers the premature stop codon of its parent TNG67, resulting in full-length translation. The ATG and TGA represent the start and stop codon of *Pi9-169*, respectively. (**b**) TG-1, TG-2, and TG-3, The gene-edited plants which reversed the early stop mutation of TNG67 as read-through reading frame. TG-4, TG-5, and TG-6, The gene-edited plants with knock-out of NBS-LRR2 in SA0169. The results do not support that the NBS-LRR2 is the causal gene responsible for the blast resistance of SA0169, and suggested that other R candidates within the *Pi169-6*(*t*) shall be responsible for the blast resistance in SA0169. Blast isolate, EM1a1-1903.
