# Supplementary material for: Two genomic regions of a sodium azide induced rice mutant confer broad-spectrum and durable resistance to blast disease
Source: Rice (N Y). 2022 Jan 10;15:2. doi: 10.1186/s12284-021-00547-z (PMC8748607; doi:10.1186/s12284-021-00547-z)
Supplement: Supplementary file 12 — Additional file 12: Figure S3. The breeding scheme of the lines with blast and bacterial blight double resistance [file 12284_2021_547_MOESM12_ESM.docx]

| 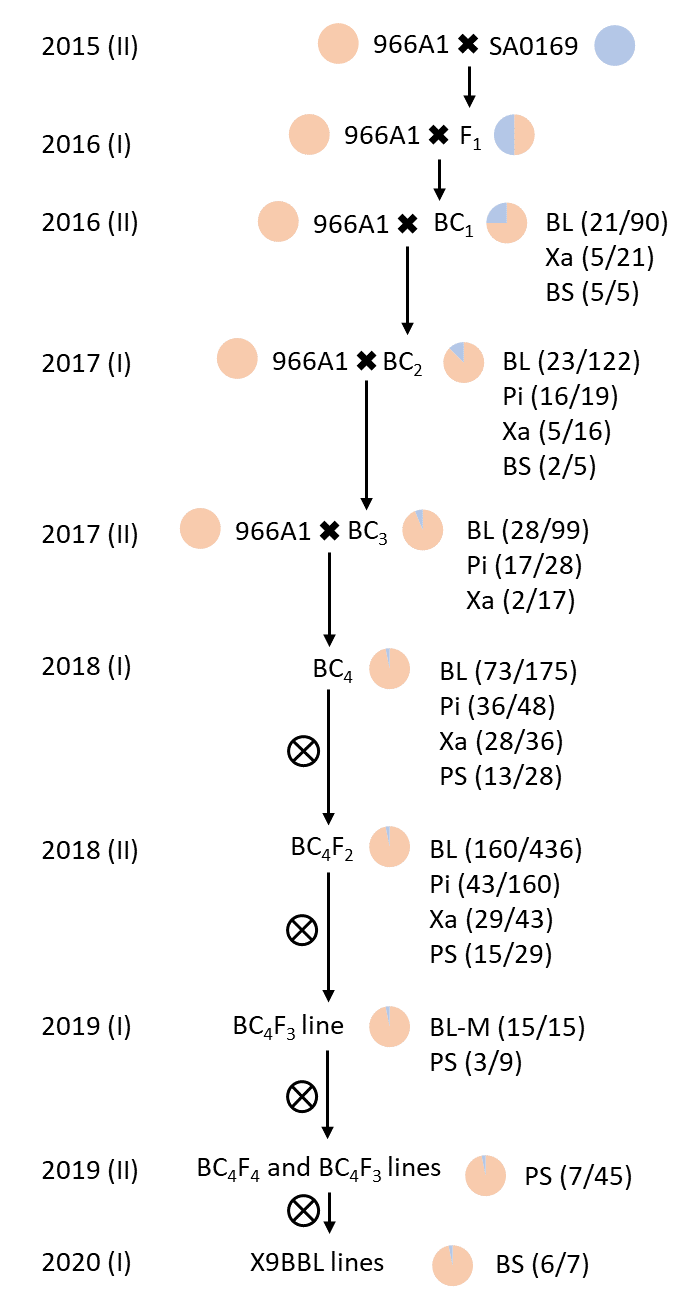 |
| --- |

**Fig. S3** The breeding scheme of the lines with blast and bacterial blight double resistance. BL, selection by using mixed blast isolates challenge; Xa, marker-assisted selection (MAS) of *Xa* genes; BS, background selection; Pi, MAS of *Pi169-6*(*t*) and/or *Pi169-11*(*t*); PS, phenotypic selection; BL-M, Selection by using MS2a2-1209 isolate
