# Supplementary material for: Two genomic regions of a sodium azide induced rice mutant confer broad-spectrum and durable resistance to blast disease
Source: Rice (N Y). 2022 Jan 10;15:2. doi: 10.1186/s12284-021-00547-z (PMC8748607; doi:10.1186/s12284-021-00547-z)
Supplement: Supplementary file 13 — Additional file 13: Figure S4. Disease resistance study against rice bacterial blight (BB) pathogen Xanthomonas oryzae pv. oryzae (Xoo) XF-89b strain [file 12284_2021_547_MOESM13_ESM.docx]

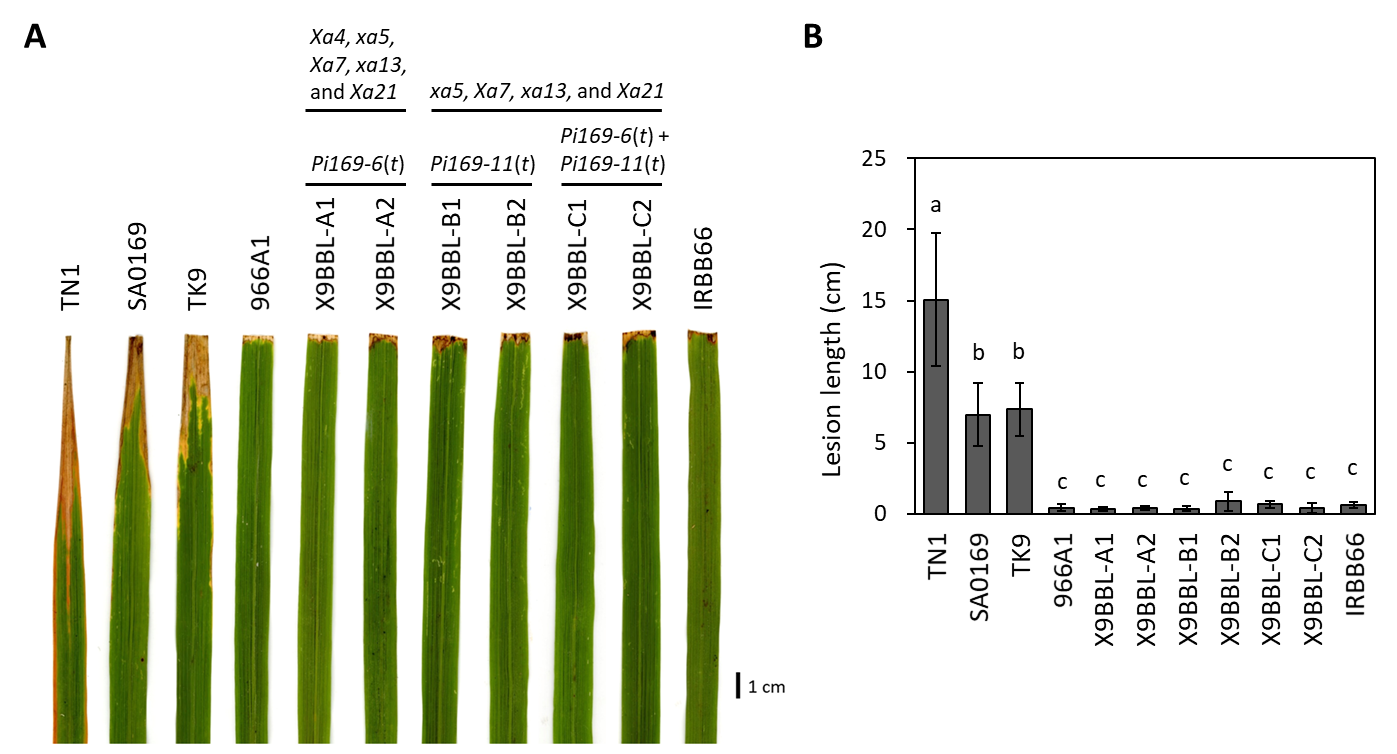


**Fig. S4** Disease resistance study against rice bacterial blight (BB) pathogen *Xanthomonas oryzae* pv. *oryzae* (*Xoo*) XF-89b strain. (A) Photographs were taken at 30 days after inoculation. TN1 (Taichung Native 1), BB highly susceptible variety; IRBB66, BB resistant line with 5 *Xa* genes (*Xa4*, *xa5*, *Xa7*, *xa13*, and *Xa21*); SA0169, blast (BL) resistant line with two mapped *R* regions (*Pi169-6*(*t*) and *Pi169-11*(*t*)); TK9 (Taikeng 9), a famous Taiwan commercial variety; 966A1, BB resistant line on the TK9 background with 5 *Xa* genes (*Xa4*, *xa5*, *Xa7*, *xa13*, and *Xa21*) and used as the recurrent parent of the NILs; X9BBL-A1, -A2, -B1, -B2, -C1, and -C2 are the BL and BB double resistant NILs. The genotypes of each NILs are shown in above. (B) Multiple comparisons were conducted and the different letters are significantly different at 5% level by Fisher’s protested LSD (Least Significant Difference) test.
