# Supplementary material for: Two genomic regions of a sodium azide induced rice mutant confer broad-spectrum and durable resistance to blast disease
Source: Rice (N Y). 2022 Jan 10;15:2. doi: 10.1186/s12284-021-00547-z (PMC8748607; doi:10.1186/s12284-021-00547-z)
Supplement: Supplementary file 14 — Additional file 14: Figure S5. Plant morphology of the newly developed blast and bacterial blight double resistant lines [file 12284_2021_547_MOESM14_ESM.docx]

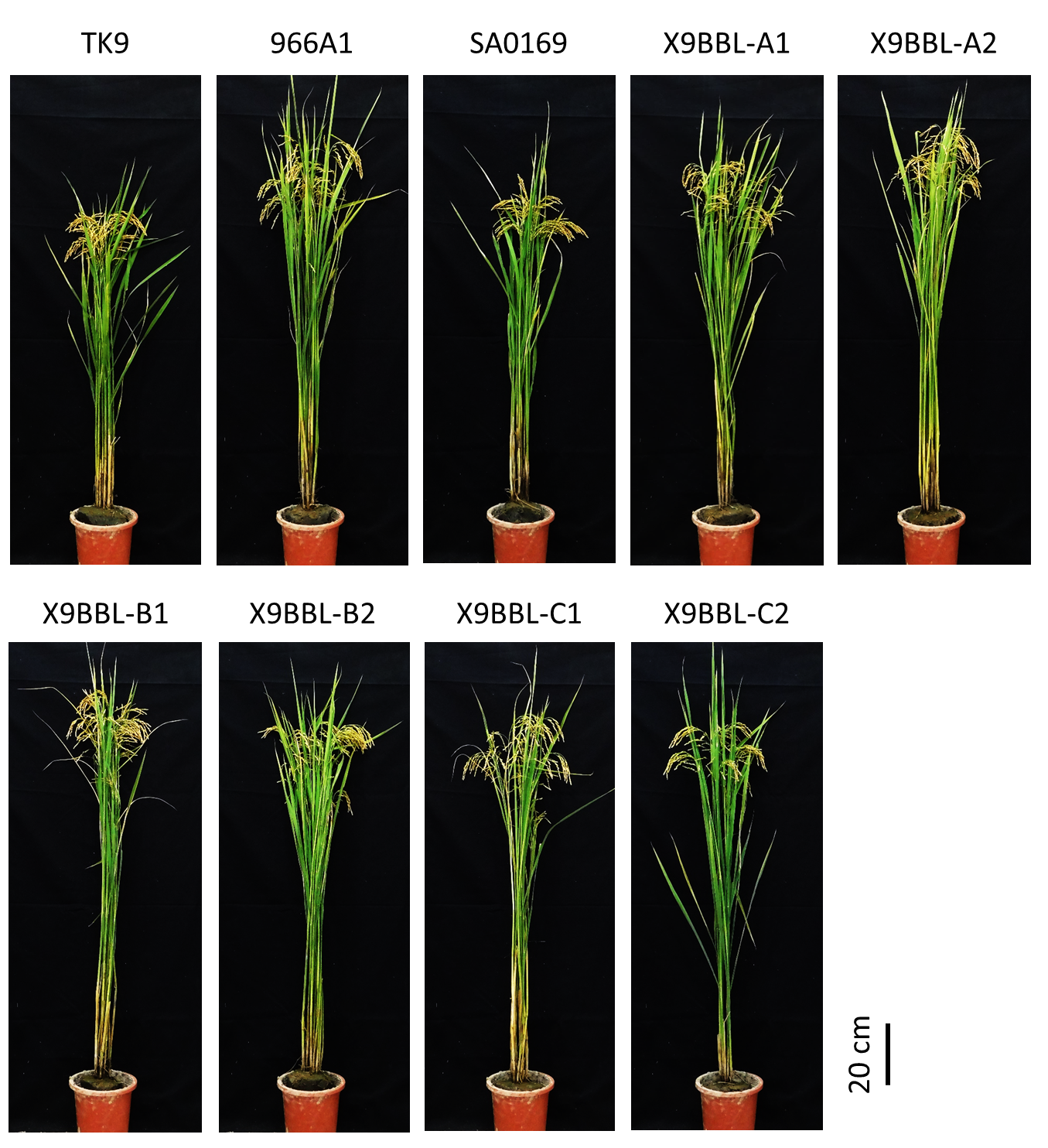


**Fig. S5** Plant morphology of the newly developed blast and bacterial blight double resistant lines. TK9 (Taikeng 9), a famous Taiwan commercial variety; 966A1, broad-spectrum bacterial blight (BB) resistant line on the TK9 background with 5 *Xa* genes (*Xa4*, *xa5*, *Xa7*, *xa13*, and *Xa21*) and used as the recurrent parent of the NILs; SA0169, sodium azide induced broad-spectrum blast (BL) resistant mutant (BL resistant donor); X9BBL-A1, -A2, -B1, -B2, -C1, and -C2 are the BL and BB double resistant NILs.
